# 1730. Increasing Vaccination Rates of 23-valent Pneumococcal Polysaccharide Vaccine Among Patients at High-Risk for Invasive Pneumococcal Disease

**DOI:** 10.1093/ofid/ofad500.1562

**Published:** 2023-11-27

**Authors:** Edward Lyon, Tracey Wetzel, Ann Wirtz, Doug Swanson, Rachel R Moran, Jessica Peters, Christine A Symes, Liset Olarte, Rana E El Feghaly

**Affiliations:** Children's Mercy Hospital, Kansas City, Missouri; Children's Mercy Hospital, Kansas City, Missouri; Children's Mercy Kansas City, Kansas City, Missouri; Children's Mercy, Kansas City, Missouri; Children's Mercy Hospital, Kansas City, Missouri; Children's Mercy Hospital, Kansas City, Missouri; Children's Mercy Hospitals and Clinics, Kansas City, Missouri; Children's Mercy Kansas City, Kansas City, Missouri; Children's Mercy Kansas City, Kansas City, Missouri

## Abstract

**Background:**

Pneumococcal disease causes significant morbidity and mortality in children. Routine childhood immunizations protect for 13 or 15 pneumococcal serotypes via two pneumococcal conjugated vaccines. Patients with immunocompromising and chronic medical conditions are at high risk of invasive pneumococcal disease. A 23-valent pneumococcal polysaccharide vaccine (PPSV23) is recommended in these patients to protect against more pneumococcal serotypes, but many patients have not received it. Our AIM is to increase PPSV23 vaccination rates among eligible patients in both the inpatient and outpatient infectious diseases (ID) settings from a baseline of 44% to 55% by October 2024.

**Methods:**

In collaboration with Children’s Mercy integrated care solutions, we created a report of eligible patients evaluated by ID in the inpatient and outpatient settings. This report captures pneumococcal immunization history of patients eligible for PPSV23. In October 2022, we formed a multi-disciplinary team of ID nurses, a pharmacist, providers, and a patient advocate. We identified several potential causes of low PPSV23 vaccination rate and developed countermeasures (figure 1). Since October 2022, we have discussed the project weekly during our divisional huddle. In January 2023, we matched outpatient and inpatient reports to include maximal information and created a 2-way communication strategy where outpatient nursing staff notifies ID providers of patients who qualify for PPSV23. In February 2023, we created an EMR shared phrase to include in ID provider notes and a badge buddy with qualifying conditions for PPSV23 vaccination.

**Figure:**
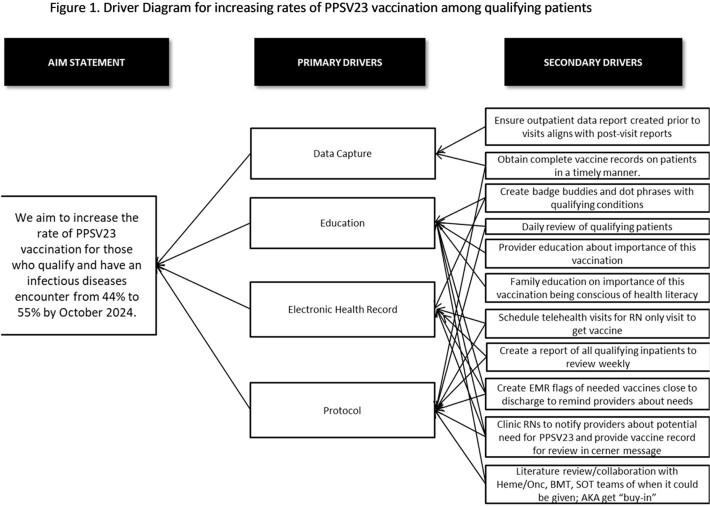

**Results:**

Following the initiation of the project we have seen an increase in qualifying patients who have received PPSV23 that has so far been sustained for 6 months (48-56%); we also see narrowing of the control limits implying improved process (figure 2).

**Figure:**
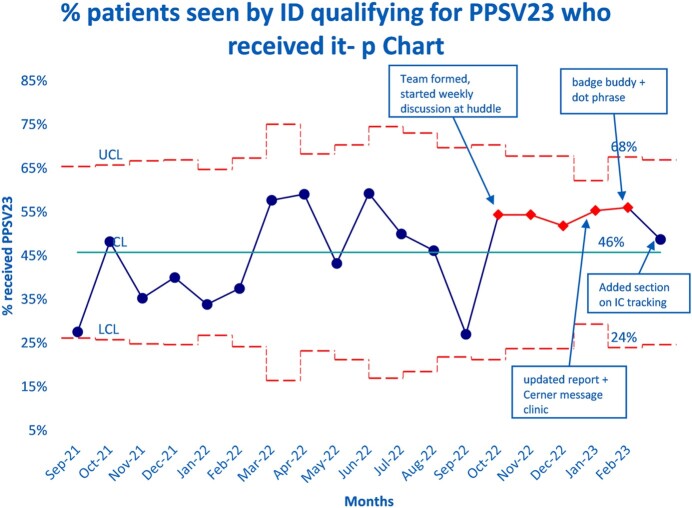

**Conclusion:**

We were able to increase PPSV23 vaccination of eligible high-risk patients in both the outpatient and inpatient settings. If this trend is sustained, we will be able to move our center line in 2 months. We have multiple additional plan-do-study-act cycles planned over the next several months to include electronic medical record changes, collaboration with different specialties and family education.

**Disclosures:**

**Doug Swanson, MD**, Merck: Grant/Research Support|Sanofi: Grant/Research Support **Liset Olarte, MD, MSc**, GSK: Grant/Research Support|Merck: Grant/Research Support|Pfizer: Grant/Research Support|Sanofi: Grant/Research Support

